# Pathways systematically associated to Hirschsprung’s disease

**DOI:** 10.1186/1750-1172-8-187

**Published:** 2013-12-02

**Authors:** Raquel M Fernández, Marta Bleda, Berta Luzón-Toro, Luz García-Alonso, Stacey Arnold, Yunia Sribudiani, Claude Besmond, Francesca Lantieri, Betty Doan, Isabella Ceccherini, Stanislas Lyonnet, Robert MW Hofstra, Aravinda Chakravarti, Guillermo Antiñolo, Joaquín Dopazo, Salud Borrego

**Affiliations:** 1 Department of Genetics, Reproduction and Fetal Medicine, Institute of Biomedicine of Seville (IBIS), University Hospital Virgen del Rocío/CSIC/University of Seville, Av. Manuel Siurot s/n, Seville, 41013, Spain; 2 Centre for Biomedical Network Research on Rare Diseases (CIBERER), Valencia, Spain; 3 Department of Computational Genomics, Centro de Investigación Príncipe Felipe (CIPF), c/Eduardo Primo Yufera, 3, Valencia, 46012, Spain; 4 Center for Complex Disease Genomics, McKusick-Nathans Institute of Genetic Medicine, Johns Hopkins University School of Medicine, Baltimore, MD, USA; 5 Department of Medical Genetics, University of Groningen, Groningen, The Netherlands; 6 INSERM U-781, AP-HP Hôpital Necker-Enfants Malades, Paris, France; 7 Laboratorio di Genetica Molecolare, Istituto Gaslini, Genova, Italy; 8 Functional Genomics Node (INB), CIPF, Valencia, Spain

## Abstract

Despite it has been reported that several loci are involved in Hirschsprung’s disease, the molecular basis of the disease remains yet essentially unknown. The study of collective properties of modules of functionally-related genes provides an efficient and sensitive statistical framework that can overcome sample size limitations in the study of rare diseases. Here, we present the extension of a previous study of a Spanish series of HSCR trios to an international cohort of 162 HSCR trios to validate the generality of the underlying functional basis of the Hirschsprung’s disease mechanisms previously found. The Pathway-Based Analysis (PBA) confirms a strong association of gene ontology (GO) modules related to signal transduction and its regulation, enteric nervous system (ENS) formation and other processes related to the disease. In addition, network analysis recovers sub-networks significantly associated to the disease, which contain genes related to the same functionalities, thus providing an independent validation of these findings. The functional profiles of association obtained for patients populations from different countries were compared to each other. While gene associations were different at each series, the main functional associations were identical in all the five populations. These observations would also explain the reported low reproducibility of associations of individual disease genes across populations.

## Background

Unlike a minority of Mendelian traits, most human diseases have complex, multifactorial inheritance where the causation resides in small allelic differences in many genes occurring in a complex manner. For these phenotypes, onset, penetrance, recurrence risk, etc., are features not dependent on one single gene, but are rather emergent properties of the ensemble of genotypes at many loci [1]. A representative example of this kind of trait is Hirschsprung’s disease (HSCR, OMIM 142623), a neurocristopathy characterized by the absence of intramural ganglion cells in the myenteric and submucosal plexuses along a variable portion of the distal intestine. Based on the length of the aganglionic region, the disorder is classified into short segment (S-HSCR: aganglionosis up to the upper sigmoid colon, 80% of cases), long-segment (L-HSCR: aganglionosis up to the splenic flexure and beyond, 17% of cases) and total colonic aganglionosis forms (TCA, 3% of cases). The most widely accepted etiopathogenetic hypothesis for HSCR is based on a defect of craniocaudal migration of neuroblasts originating from the neural crest that, under normal circumstances, reach the small intestine in the week 7 of gestation and the rectum in the 12th week [2,3]. HSCR constitutes a complex pathology with non-Mendelian inheritance, sex-dependent penetrance, variable expression and suggestive of the involvement of one or more gene(s) with low penetrance [2,3]. With a relative risk as high as 200, HSCR can be considered an excellent model to study common multifactorial diseases. The major HSCR predisposing event is the presence of a haplotype at the *RET* proto-oncogene [4,5] (OMIM 164761, 10q.11) which comprises a SNP lying in an enhancer element of the intron 1 [2,3,6-8]. To date, several HSCR-associated regions, such as 10q11 [9-12], 13q22 [10], 9q31 [9,13], 3p21 [11,14], 19q12 [11], 16q23 [10], 21q21 [12], 4q31.3-q32.3 [15] or 8p12 [16], have been described. Moreover, in some cases, the HSCR gene within the associated region has been already identified, as it is the case of *RET* at 10q11, *EDNRB* (OMIM 131244) at 13q22, or *NRG1* (OMIM 142445) within 8p12 [16]. In addition, a very recent study based on pathways and networks analyses of a Spanish series of HSCR patients, described associations of four new loci (*RASGEF1A*, *IQGAP2*, *DLC1* and *CHRNA7*) to the disease [17].

Conventional gene-based association tests present obvious limitations, especially in the context of rare diseases, where the recruitment of large cohorts of patients is extremely difficult. However, Pathway-based analysis (PBA) strategies [18-20], which allows for the detection of modules of functionally-related genes associated to the disease, have already been successfully applied to the study of a number of diseases [17,21-23]. The recent description of HSCR-associated functional modules in Spanish population [17] constitutes an excellent example of how a PBA strategy can be successfully applied to define the molecular basis of the mechanism of the disease. Here we have extended such approach to different populations of the International Consortium for Hirschsprung disease (ICHSCR) which have provided samples from France, Italy, the Netherlands and the USA. The extended study allowed us to conclude that functional modules related to signal transduction and its regulation, neurogenesis and the Ras pathway are common to all the populations in spite of the fact that the most associated genes in each population were different. Moreover, Network analysis recovers sub-networks significantly associated to the disease and populated by genes with the same functionalities, thus confirming the findings of the PBA analysis by an independent methodology.

## Methods

### General analysis strategy

**Figure 1:**
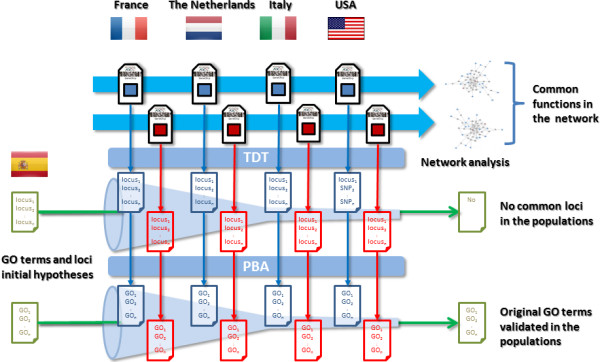
**Schema of the analysis strategy used to validate the findings in the Spanish population [**[17]**] with the available scattered information about the four additional populations present in the consortium.**

Based in the recent study which identified four new loci associated to HSCR [17] as a starting point, we aimed to validate whether the discoveries made were specific for the analysed population or, on the contrary, were common mechanisms shared by other populations. Figure 1 shows the strategy followed in this study: our initial hypothesis was that disease genes would probably be population specific whereas functional modules would be universal.

### Samples and SNP genotyping

We have conducted a genome wide genotyping of a multinational cohort of 162 trios of sporadic short-segment Hirschsprung’s patients recruited in the context of the ongoing initiative of the ICHSCR. Genotyping was carried out using the Affymetrix 500 k chip (composed of the 250 k *Nsp* and the 250 k *Sty* chips) (Table 1). Given that not all the trios were simultaneously genotyped with both chips yet (only 26 of them), we considered two independent measurements, one for each chip (*Sty* and *Nsp*, with 97 and 91 trios, respectively), of the samples analyzed. Quality controls were as follows: SNPs missing in more than 20% of the samples in the calling process, SNPs with MAF < 0.5%, with Mendelian errors or not in Hardy-Weinberg equilibrium (in unaffected samples; p-value < 10^-5^) and samples with more than 5% of the SNPs missing were discarded. Finally, incomplete families (trios) were also discarded. Additional file 1: Table S1 summarizes the effects of the different steps of the quality control.

**Table 1:** Available trios for any of the chips distributed among the five country populations analyzed

**Chip**	**France**	**Italy**	**USA**	**Netherlands**	**Total**
***Nsp***	15	20	25	37	97
***Sty***	16	18	25	32	91
***Nsp*** **+** ***Sty***	5	1	9	11	26
**Total trios**	26	37	41	58	**162**

First and second rows refer to the trios genotyped with each specific chip (*Nsp* or *Sty*). Third file contains the trios simultaneously genotyped with both chips (*Nsp* + *Sty*). Forth row is the total number of trios used in the study for each population.

### Pathway-based analysis

We conducted a Transmission Disequilibrium Test (TDT) association analysis as implemented in the PLINK [24] software for the different sets of trios analyzed (see Figure 1).

The SNPs were ranked according to their p-values obtained in the TDT test and then a PBA test [18], as implemented in the GESBAP [25] module of the Babelomics [26] software, was conducted. PBA seeks for gene sets (GO terms in this study) associated to low p-values. This association is found significant when a number of genes of the GO term, larger than expected by chance, simultaneously display low (although not necessarily significant) individual p-values [18]. Given that only genes can be related to GO terms, PBA tests use only SNPs mapping on genes, or in the close neighborhood, here defined as 500 bps up-and downstream of the gene limits. When multiple SNPs map onto the same gene, the SNP with lowest (most significant) p-value is retained. In this way a list of genes ranked by the best of the p-values of all the SNPs mapping onto them is constructed. Then, GO terms significantly overrepresented among the genes associated to low p-values are found upon the application of a logistic regression. GO terms are declared significantly associated to HSCR when a number of its genes, larger than expected purely by chance, display a low p-value (i.e., are on the top of the list ranked by significance). To control the number of false positives due to multiple-testing effects, only GO terms with a FDR-adjusted [27] p-value < 0.05 are declared significant. The adjustment process takes into account that four populations and two chips have been tested, so all the individual tests were considered here. This is a widely accepted method for correcting p-values that account for multiple testing by controlling the rate of false discoveries. By default the GESBAP software only analyses GO terms between levels 3 and 13 of the GO hierarchy and exclude GO terms with more than 600 genes or with less than 5 genes. In this way GO terms which are either too specific or too unspecific, and only contribute to the decrease of the statistical power of the test, are avoided.

### Network analysis

Like in the PBA approach, a list of genes ranked from low to high p-values is explored in order to discover subnets with connectivity values higher than expected by chance. Briefly, the N (10 in this case) most significant genes are mapped onto the interactome and the minimum network connecting them is obtained. The connectivity of such sub-network is calculated as the average of all the individual connectivity values of all the connected genes. The connectivity parameter accounts for the number of partners of direct interaction that a particular node has. An empirical distribution of the random expectation of this parameter can be obtained by repeatedly sampling random sets of N genes from the complete genome and calculating the average connectivity of their corresponding minimum connecting trees. Thus, the real value obtained for the N most associated genes can be contrasted with respect to its random empirical expectation. If the connectivity is not significantly higher than its random expectation we repeat the procedure for the N + 1 most significant genes. The procedure is repeated until a sub-network of significant connectivity is found or a value of N too high is reached (200 in this case). This procedure [28] is a generalization of the network analysis methodology applied to the study of networks contained in gene expression signatures [29,30]. An implementation of the procedure can be found in the Babelomics package [26].

### ENCODE information for extra Genic SNPs

Extra genic SNPs have been used to find extra support for the functionalities found as associated to HSCR. We have used the HaploReg [31] tool to retrieve the relevant information from the ENCODE project [32] corresponding to the chromosomal regions in which significant extra-genic SNPs map.

## Results

### Validation of the known HSCR-associated functionalities by PBA of the available chips from the different country populations

As described in the Methods section (see Figure 1), we used the GO terms already described as part of the disease mechanism in the Spanish population [17] as the initial hypothesis that should be validated using all the available information in the four populations of the consortium. Then, an independent PBA was carried out for each chip in each population. The p-values of the SNPs in each chip/population were obtained by means of a TDT test, as implemented in the PLINK package. Such p-values were introduced in the PBA section of the Babelomics package to complete the PBA test and obtain lists of GO terms significantly associated to each condition tested.

Results are summarized in Table 2. All the GO terms previously proposed as components of the disease mechanism were validated in the four populations in one or both chips, with the exception of *enzyme linked receptor protein signaling pathway* (GO:0007167), which seems to be an aspect of signaling peculiar of the Spanish population. In addition to the proposed GO terms other new terms have been included in Table 2 because of their consistent significance across the populations analyzed and also because of their relationship to ENS formation. However, it is known that gene-based p-values are biased towards longer genes and those within weak LD region. Such genes have higher probability of encompassing more independent SNPs that will increase the probability of displaying significant p-values just by chance [33]. Although this bias is less expectable in GO terms, because these are composed by numerous genes with no *a priori* bias towards any particular size, it is true that they exist GO terms with a unexpectedly high number of large genes, whose p-values can be biased [34]. In order to check potential biases due to abnormally number of large genes in GO terms we have plotted the distribution of gene sizes of each significant class in Table 2 against the background distribution of sizes and only three of them were slightly bigger that the background distribution (see Additional file 2: Figure S1). Actually, three terms show a median size, which is higher than the 3rd quartile of the distribution of median gene sizes of all the GO terms (see Additional file 3: Figure S2). These GOs, however, are biologically related to the disease and related to other GOs without this size bias.

**Table 2:** **GO modules significantly associated to HSCR in the different populations analyzed with the *Nsp *** **and *Sty *** **chips, separately**

	**GO ID**	**Initial hypothesis**	**Italy**		**France**		**Netherlands**		**USA**	
			***Nsp***	***Sty***	***Nsp***	***Sty***	***Nsp***	***Sty***	***Nsp***	***Sty***
Signaling	GO:0051056	Regulation of small GTPase mediated signal transduction	Y		Y		Y		Y	Y
	GO:0046578	Regulation of Ras protein signal transduction	Y		Y*		Y		Y*	Y
	**GO:0007265**	**Ras protein signal transduction**	**Y**		**Y**		**Y**		**Y**	**Y**
	**GO:0007264**	**Small GTPase mediated signal transduction**	Y*		Y*		Y*		Y*	
	GO:0035023	Regulation of Rho protein signal transduction	Y		Y		Y*			
	**GO:0009966**	**Regulation of signal transduction**	Y*		Y*		Y*			**Y**
	**GO:0007167**	**Enzyme linked receptor protein signaling pathway**							**Y**	
	**GO:0007268**	**Synaptic transmission**	**Y**		**Y**		**Y**		**Y**	
ENS formation	**GO:0006816**	**Calcium ion transport**			**Y**	**Y**	Y*		Y*	Y*
	**GO:0006812**	**Cation transport**		Y*	**Y**	Y*	**Y**		Y*	Y*
	**GO:0016337**	**Cell-cell adhesion**	Y*	**Y**	**Y**		**Y**		**Y**	
	*GO:0016477*	*Cell migration*	*Y*	*Y*			*Y*			
	*GO:0007399*	*Nervous system development*	*Y*		*Y*		*Y*		*Y*	
	*GO:0048666*	*Neuron development*	*Y*		*Y*		*Y*			
	*GO:0007409*	*Axonogenesis*	*Y*		*Y*		*Y*			

The first column specifies the general biological process represented by the GO terms (columns 2 and 3) which were found significant in the Spanish population and constitute the initial hypothesis for the functional basis of the disease. From fourth column ahead a Y means that the GO term was significant in the corresponding population and chip (FDR-adjusted p-values < 0.05) using the PBA approach described in Methods. Y* means that although this particular term was not significant, another direct descendant or ancestor GO term in the hierarchy was significant. The last four files are GO terms (in italics) that were not initially significant in the Spanish population but were consistently significant through the populations analyzed here.

**Figure 2:**
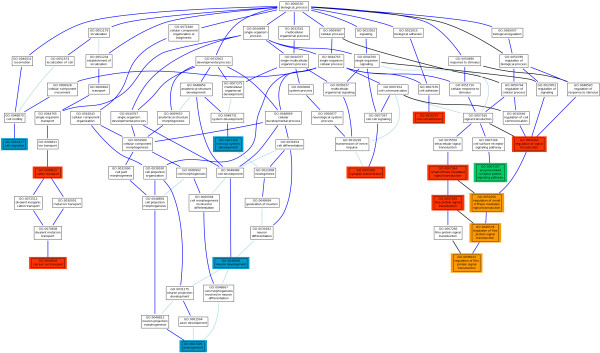
**Hierarchy of GO terms significantly associated to HSCR in the different populations analyzed with the *Nsp *and *Sty *chips, separately (See Table**2**).** Red color means that the GO term, originally significant in the Spanish population [17] was also significant in all the four Consortium populations in at least one of the chips (FDR-adjusted p-values < 0.05) using the PBA approach described in Methods. In Orange color are represented those initially red color GO term in which there seems to be a potential bias due to the size of the genes composing the GO. In green color there is a GO term, significant in the Spanish population and significant in only one population of the consortium (USA). The blue color correspond GO terms that were not initially significant in the Spanish population but were consistently significant through the populations analyzed here.

Figure 2 graphically summarizes a comprehensive map of the biological functionalities associated to the disease. GOs potentially affected by gene size bias are represented in a different color. As previously described, GO modules related to signal transduction and its regulation that include the parent *regulation of signal transduction* (GO:0009966) and the rest of descendants (*regulation of small GTPase mediated signal* transduction, GO:0051056; *regulation of Ras protein signal transduction*, GO:0046578; and *regulation of Rho protein signal transduction*, GO:0035023), potentially affected by gene size bias, and the regulatory terms small GTPase mediated signal transduction (GO:0007264) and *Ras protein signal transduction* (GO:0007265). However, enzyme linked receptor protein signaling pathway (GO:0007167) was only confirmed by the USA population, suggesting that it is not a general mechanism of the disease but it rather represents a population-specific peculiarity. Finally, *synaptic transmission* (GO:0007268) a process whose malfunction is licit to consider to have a role in the disease, has also been validated in all the studied populations. On the other hand, cation transport (GO:0006812) and, in particular, the descendent term calcium ion transport (GO:0006816) are known to be altered in the disease. Both terms have been validated in the four populations analyzed. Finally, another functionality affected in all the populations, related to the formation of functional enteric cells, is *cell-cell adhesion* (GO:0016337). Of particular interest is the discovery of a series of GO terms involved in the generation of neurons that take part of the ENS, such as *nervous system development* (GO:0007399) and the descendant terms *neuron development *(GO:0048812) and *axonogenesis* (GO:0007409). These terms did not reach a significant p-value in the Spanish populations [17] but, no doubt these functionalities can be considered as part of the underlying mechanism of the disease.

### Comparison of the gene functionalities associated to HSCR observed between country sample populations

Apart from the validation of the core disease related functionalities, the independent study of the functional associations obtained by PBA of the 4 different country populations of the ICHSCR rendered a considerable number of GO terms.

Again, when individual country populations are independently analysed two key processes become apparent: signalling and neurogenesis. In fact, the GO terms *synaptic transmission*, *regulation of small GTPase mediated signal transduction, Ras protein signal transduction* and *nervous system development* were significant in all the 5 analysed populations. Moreover, it is clear from the figures depicting the GO structure (Additional file 4: Figure S3 and Additional file 5: Figure S4) that a relevant number of GO subcategories not shared by all the country populations actually belong to three main branches: nervous system development, signal transduction and cell migration. Additional file 6: Table S2 and Additional file 7: Table S3 show a total of 49 and 29 GO modules, respectively, significantly associated to HSCR found in the analysis individualized by population in any of the two chips. Additional file 4: Figure S3 and Additional file 5: Figure S4 show how all these modules are inter-related among them. For example, terms like *axonogenesis, neuron development, neuron differentiation, generation of neurons*, are all descendants of *neurogenesis*, which is itself, a descendant of *nervous system development. Cell-cell adhesion* is also a parent term of many other GO terms related with the formation of functional enteric cells. And there is also the case of other processes whose malfunction is relevant in the disease, such as *synaptic transmission*, the transport of several substances (*cation transport* or *phospholipid transport*) or different signalling-related functions, including the well-known RAS/RHO intracellular signalling pathway.

**Figure 3:**
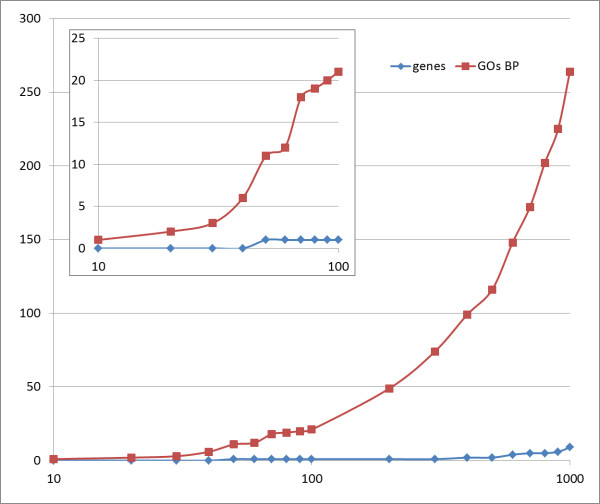
**Number of genes (blue line) and GO terms common to the four Consortium populations studied and the Spanish population.** The X axis (in logarithmic scale) represents the list of SNPs ranked by p-value. For example, within the 50 first SNPs there is only one gene common to the five populations, *AGAP3*, while SNPs in different genes were defining 11 GO terms common to the five populations analyzed. The upper left square represents a detailed view of the first 100 ranked SNPs.

In order to know if the GO module associations found in each population were consistently a consequence of the underlying associations of the same specific genes, we compared the individual gene association values across all country populations. A list with the SNPs mapping within or in the neighborhood of genes ranked by association to HSCR (that is, the p-value of the TDT) was generated. Figure 3 shows the genes common to the four Consortium populations analyzed, plus the Spanish population already studied [17]. Only *AGAP3* and *TUBA8* genes are common to the five populations when the 400 SNPs showing the highest association to HSCR are considered. However, when the GO terms corresponding to the genes are compared across populations, a remarkable coincidence of affected functions occurs, as reflected in Table 2. While in the case of genes we need to reach up to 200 SNPs to have more than one common gene, and up to 500 SNPs to have a third common gene (apart from *AGAP3* and *TUBA8*), when GO terms corresponding to the genes that appear in the rank of the first 10 SNPs are compared many coincidences are found. If we expand the percentile, the coincidences in functionalities increase enormously. All the coincident functionalities are related again to signaling and ENS formation.

Therefore, our observations strongly suggest that common functions affected in different populations are not due to common genes but rather to different genes of common functionality, which are affected in different populations and cause similar phenotypic effects.

### Network analysis

**Figure 4:**
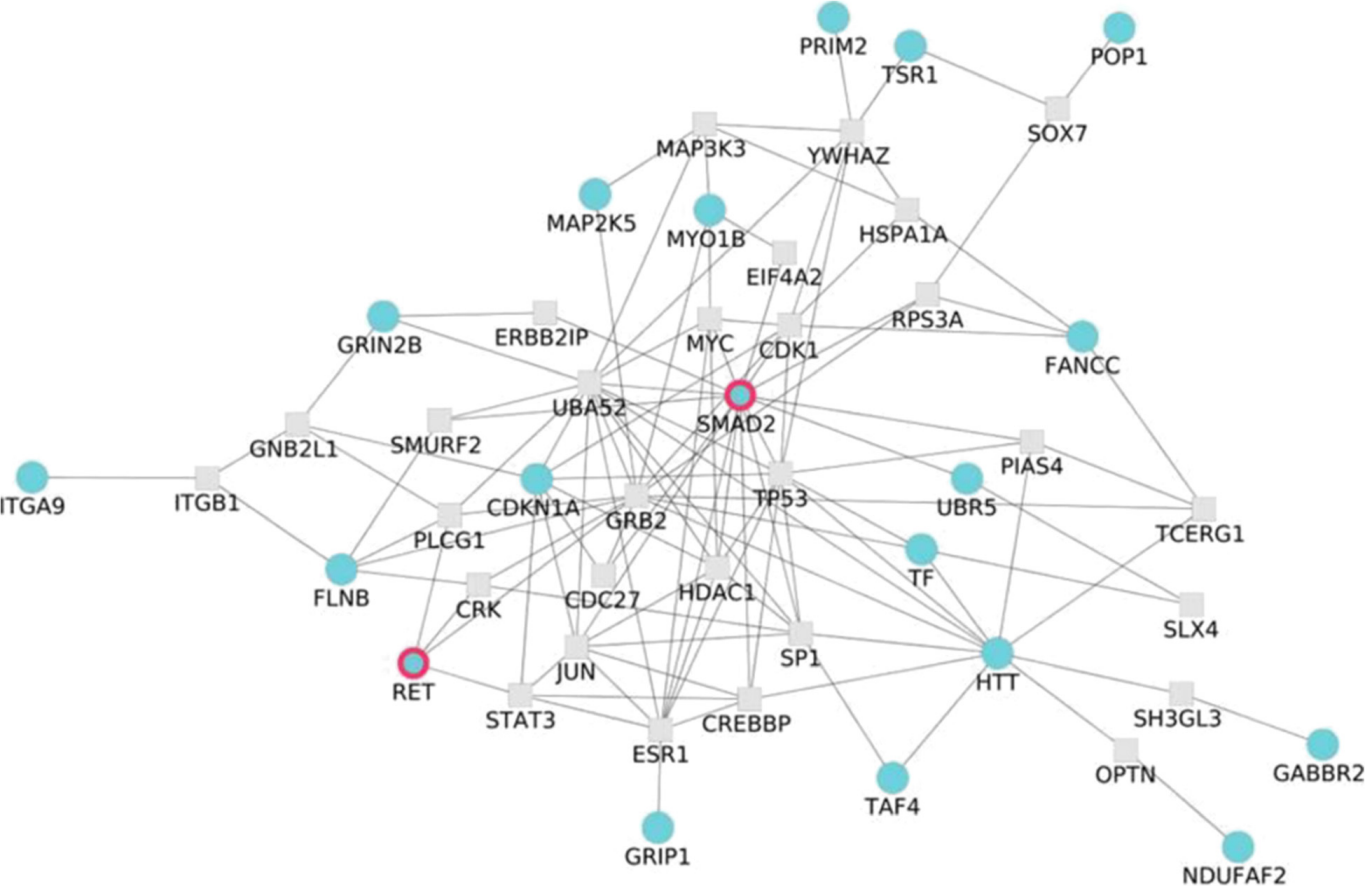
**Significant sub-network of 53 genes, allowing for one intermediate gene, associated to HSCR (p-value = 0.027) in the *Nsp *chip.** The network analysis [28] is carried out by using the functional analysis options (Set enrichment analysis/NetworkMiner) of Babelomics [26] on the list of genes ranked by the p-values obtained upon the application of a conventional TDT association test as implemented in PLINK [24].

The lists of genes from both chips, ranked by association to HSCR (according to SNP p-values obtained from the corresponding TDTs), were scanned to find sub-networks of interacting proteins significantly associated to the disease [28]. The application of this network analysis to the *Nsp* chip rendered a significant network (p-value = 0.027; see Methods) within the first 27 most associated genes, linking 19 of them. In the derivation of the network a maximum of one external node linking these proteins was allowed [28]. This significantly large sub-network (Figure 4) significantly associated to the disease documents the basis of the complexity of this disorder. Genes like *GABBR2*, *GRIN2B* and *HTT* associated to synaptic transmission (GO:0007268), and the last one also associated to ,central nervous system development (GO:0007417) and neuron development (GO:0048666) are among the 27 most associated genes, along with *RET*, also in the list. Interestingly, genes connecting them and included in the network significantly associated to the disease are: *CRK*, related with regulation of small GTPase mediated signal transduction (GO:0051056), regulation of Ras protein signal transduction (GO:0046578) and Ras protein signal transduction (GO:0007265); *GRB2*, related to Ras protein signal transduction (GO:0007265); *ITGB1*, related to cell-cell adhesion (GO:0016337), cell migration (GO:0016477), cell projection organization (GO:0030030) and neuron development (GO:0048666); *PLCG1*, related to cell migration (GO:0016477); *RPS27AP16*, related to synaptic transmission (GO:0007268), cell projection organization (GO:0030030) and neuron development (GO:0048666); *SH3GL3* and *TP53*, related to central nervous system development (GO:0007417). Again, the main processes highlighted by the network analysis are signalling and neurogenesis. Moreover, the closeness in the interactome of the HSCR genes to the genes selected by the network analysis strongly supports the relationship of the sub-network to the disease. The application of this network analysis to the *Sty* chip results in a significant network (p-value = 0.04; see Methods) within the first 67 most associated genes. In the derivation of the network a maximum of one external node linking these proteins was allowed [28] (see Additional file 8: Figure S5). This larger network contains some of the genes already linked in the *Nsp* network plus some others and point exactly to the same affected functionalities.

### Extra-genic SNPs associated to HSCR that support the functionalities found

Very recently the results of the ENCODE project have been published, allowing to assign functionalities to regions in which some extra-genic SNPs associated to HSCR were found to map. Table 3 lists six extra-genic SNPs with a nominal p-value < 10^-5^ obtained for each chip analyzing all the trios together, for which some annotation has been found. Thus, rs2435367, one of the SNPs with a lowest p-value, is mapping in a DNAse hypersensitivity region, typically related to gene expression, close to the 5′ of *RET*. The SNP rs2505526 (p-value = 8.353×10^-7^), changes a transcription factor binding site (TFBS), Pax-4, close to the *RASGEF1A* gene, related to signal transduction (particularly the Ras pathway) and ENS formation. The SNP rs16838932 (p-value = 4.459×10^-7^) maps in a ultraconserved [35] region and it is difficult to speculate any relation with the disease by itself or through the closest gene, *SLC39A10*. The SNP rs2659635 (p-value = 7.098×10^-6^) maps in a TFBS, Foxj2, in an ultraconserved [35,36] genomic region. The SNP rs4570660 (p-value = 7.744×10^-6^) maps in a DNAse hypersensitive region and, within it, in the binding site of the transcription factor Sox. Several Sox transcription factors have been linked to neural crest evolution and development [37]. Finally, rs12067906 (p-value = 9.584×10^-6^) also maps in a DNAse hypersensitive region and onto two TFBS (corresponding to Gfi1 and HLF, both related to leukemia and several related pathologies). In addition, this site is bound by the protein RAD21, involved in DNA repair.

**Table 3:** **Extra-genic SNPs with a nominal p-value < 10**^**-5 **^**mapping in regions recently annotated in ENCODE [**[32]**]**

**SNP**	**Nominal p-value**	**Adj. p-value (BH)**	**Chip**	**RefSeq genes**	**Feature**	**Proteins bound**	**Motifs changed**
rs2435367	8.067×10^-11^	2.2083×10^-06^	*Sty*	5.9 kb 5′ of **RET**	DNAse hypersensitivity		
rs2505526	8.353×10^-07^	0.01506847	*Sty*	7.5 kb 3′ of RASGEF1A			Pax-4
rs16838932	4.459×10^-07^	0.021091873	*Nsp*	421 kb 5′ of SLC39A10	Ultraconserved region [35]		
rs2659635	7.098×10^-06^	0,08204207	*Sty*	125 kb 5′ of XRCC6BP1	Ultraconserved region [35,36]		Foxj2
rs4570660	7.744×10^-06^	0,08479448	*Sty*	65 kb 3′ of APOBEC1	DNAse hypersensitivity		Sox
rs12067906	9.584×10^-06^	0,09994469	*Sty*	96 kb 5′ of RGS21	DNAse hypersensitivity	RAD21	Gfi1, HLF

In this case all the trios have been analysed together for each chip.

## Discussion

Understanding the molecular and cellular processes required for proper ENS development, and therefore the corresponding defects that lead to HSCR, requires of the knowledge of the functionalities affected by the genes affected in the disease.

The PBA strategy has already been successfully applied to the study of some traits such as coronary heart disease risk [21], bipolar disorder [22], Crohn’s disease, hypertension, rheumatoid arthritis or diabetes among others [23]. These studies led to the identification of numerous pathways implicated in disease predisposition that would have not been revealed using standard single-locus GWAS statistical analysis criteria. Many of such pathways had long been assumed to contain polymorphic genes that lead to disease predisposition. The same conclusions can be extracted from our results that reveal a clear association of GO terms connected to Ras signalling, widely known as a pathway with a key role in ENS formation.

Regarding network analysis, it exploits the information contained in the interactome with the idea that proteins close in the interaction network will have a higher probability of causing the same disease and constitutes a powerful technique to detect gene-disease associations. Network analysis has been successfully applied to discover genes in different diseases, such as ataxias [38], Huntington disease [39], schizophrenia [40] or Alzheimer’s [41]. Therefore we considered it a really useful tool to be applied to the results derived from the multinational GWAS in the context of HSCR.

The recent description of HSCR-associated functional modules in Spanish population [17] constitutes an excellent initial functional hypothesis of the molecular mechanism of HSCR. We have used all the data available of an international cohort of 162 trios of short-segment Hirschsprung’s disease to carry out a multi-population PBA study that validate the initial functional hypothesis. Specifically, our study has led us to the confirmation of a spectrum of different GOs related to the disease, being of special interest those terms related to signal transduction, such as the ones connected to Ras signaling. The Ras pathway is known to be one of the intracellular signaling mediated by the RET receptor, and is involved in cell survival and proliferation, both of them key biological processes related to ENS formation [42]. Thus, in spite of some apparent bias due to the size of the genes in these GO terms, there are solid biological basis for the involvement of such pathways in the disease. In addition, previous studies have demonstrated that signaling through the small Rho GTPases is also important for colonization of the gut by enteric neural crest cells and the concomitant growth of axons [42]. These results strongly suggest that members of the Ras/Rho protein signal transduction or regulators may play a key role in the pathogenesis of HSCR (Figure 2). Previous studies have proposed some specific genes included in these GOs as potential candidate genes for HSCR. For instance, it has been shown an under-expression of the gene *Arhgef3* in mice deficient for *RET* when compared to wildtype mice, which suggests its role in ENS formation. Interestingly, the human homologue for this gene, *ARHGEF3*, maps to 3p14, a chromosomal region previously described as a susceptibility locus for HSCR [11,43], although to date its candidature has not been further evaluated. On the other hand, migration of enteric neural crest cells in the gut wall during embryogenesis requires interactions between the migrating neural crest cells and the extracellular matrix (ECM) environment in different regions of the developing gut. It implies a key role of cell migration during enteric nervous system formation, and would support the association to HSCR obtained for several related GO terms. Therefore, all these associated GOs might provide potential candidate genes implicated in ENS formation and also the pathogenesis of HSCR.

Our findings clearly show that, while the genes most associated to HSCR are essentially different in the five analyzed populations, gene modules with common functions (GO terms) are the same. Thus, the comparative analysis of the populations is revealing two important facts: a) the GO biological processes significantly associated to the disease in the different series of the ICHSCR strongly suggest that HSCR is caused in the different populations by different particular genes belonging to the same (or related) GO modules and b) such gene modules carry out biological functions that can be assimilated (using the GO hierarchy) to neurogenesis and signaling. The network analysis also points to the same processes. In other words, although we cannot exclude the existence of some causative genes common to all the populations, which still remain undetected, our results rather point to a scenario in which HSCR is the result of different genes causing approximately the same phenotypic effects in different populations.

Additionally, the observations made with the extra-genic SNPs suggest other possible disease mechanisms for HSCR more related to regulation or DNA instability. Some of these SNPs were markers of DNA hypersensitivity regions in the neighborhood of genes such as *RET* and, moreover, some of them directly point to TFBSs. One of the SNPs was pointing out a region of binding of a protein involved in DNA repair.

## Conclusions

Independent evidences obtained from common gene functionality and from physical protein-protein interactions point to HSCR as a disease caused by variants in genes belonging to some GO modules related to neurogenesis, in particular within the context of ENS formation, and signaling. Moreover, the analysis of extra-genic SNPs in a functional context provided by the recent publication of the results of the ENCODE project [32] provides additional evidences in this direction. Interestingly, while the gene associations were different across populations the affected functionalities were always the same. This suggests that the known difficulty in validating genes in different populations [1,44,45] could be more a consequence of the multigenic nature of the disease that a sampling problem. In this scenario, the low percentage of the variance of traits explained by individual genes [46] is an obvious consequence of the fact that many complex diseases are the result of different combinations of variants that occur in different populations just by founder effects. Such different sets of variants collectively cause a malfunction of particular functional modules, which constitute the ultimate cause of the disease.

In summary, this comprehensive profile of functional modules (GO) has proven to be a useful resource for future developmental, biochemical and genetic studies. Our findings indicate that this approach can help to identify candidate genes for human disease susceptibility loci. Beyond technical considerations on the advantages of using functional modules in the analysis of genotype data, the biological pathways highlighted by our study provide insights into the complex nature of HSCR, opens new opportunities for validation of new disease genes and may help in the definition of relatively tractable targets for therapeutic intervention.

One known limitation of function-based approaches is that variants not mapping within or close to genomic elements with a functional annotation remain unused in the study. However, the recent availability of new functional domains provided by the ENCODE consortium will allow the extension of theses function-based studies (PBA, network analysis, etc.) beyond the conventional studies based on genes or known regulatory elements such as miRNAs.

## Abbreviations

ENS: Enteric nervous system; FDR: False discovery rate; GO: Gene ontology; HSCR: Hirschsprung’s disease; ICHSCR: International Consortium for Hirschsprung disease; MAF: Minor allele frequency; OMIM: On-line mendelian inheritance in man; PBA: Pathway-based analysis; SNP: Single nucleotide polymorphism; TDT: Transmission disequilibrium test; TFBS: Transcription factor binding site.

## Competing interests

The authors declare that they have no competing interests.

## Authors’ contributions

RMF, BL-T, SB, GA and JD drafted the manuscript. SA, YS, CB, FL, BD, IC, SL, RMWH, AC produced the ICHSCR data. MB and LGA carried out the PBA and the network analyses. JD conceived and coordinated the data analysis. SB conceived the study and coordinated all the laboratory tasks. All authors read and approved the final manuscript.

## Supplementary Material

Additional file 1: Table S1 Summary of the effects of the different steps of the quality control. Click here for file

Additional file 2: Figure S1 Gene length distribution of gene lengths within GO terms. Significant GO terms from Table 2 are plotted in blue. The background distribution of gene lengths in the rest of non-significant GO terms is represented in yellow. Click here for file

Additional file 3: Figure S2 Boxplots of gene length distribution of gene lengths within GO terms. The first boxplot on the left, in yellow, represents the distribution of genes in all the non-significant GO terms. The rest of boxplots in blue correspond to the significant GO terms from Table 2. Click here for file

Additional file 4: Figure S3 Tree hierarchy depicting the relationships between GO terms significantly associated to HSCR (FDR adjusted p-value < 0.05) using the PBA [25] as implemented in Babelomics [26] in the four country populations of the Consortium: French, Italian, Dutch and USA for the *Nsp* chip. The results previously obtained for the Spanish population [17] have also been added. Significant terms have been color-coded according the number of populations in which the GO terms was found to be significant. The darkest values corresponds to GO terms significant in five populations and the palest in only one population (see Additional file 6: Table S2). Click here for file

Additional file 5: Figure S4 Tree hierarchy depicting the relationships between GO terms significantly associated to HSCR (FDR adjusted p-value < 0.05) using the PBA [25] as implemented in Babelomics [26] in the four country populations of the Consortium: French, Italian, Dutch and USA for the *Sty* chip. The results previously obtained for the Spanish population [17] have also been added. Significant terms have been color-coded according the number of populations in which the GO terms was found to be significant. The darkest values corresponds to GO terms significant in two populations and the palest in only one population (see Additional file 7: Table S3). Click here for file

Additional file 6: Table S2 GO modules significantly associated to HSCR (FDR adjusted p-value < 0.05) using the PBA [25] as implemented in Babelomics [26] found in the analysis individualized by population in the *Nsp* chip. Click here for file

Additional file 7: Table S3 GO modules significantly associated to HSCR (FDR adjusted p-value < 0.05) using the PBA [25] as implemented in Babelomics [26] found in the analysis individualized by population in the *Sty* chip. Click here for file

Additional file 8: Figure S5 Significant sub-network of 65 genes, allowing for one intermediate gene, associated to HSCR (p-value = 0.04) in the *Sty* chip. The network analysis [28] is carried out by using the functional analysis options (Set enrichment analysis/NetworkMiner) of Babelomics [26] on the list of genes ranked by the p-values obtained upon the application of a conventional TDT association test as implemented in PLINK [24]. Click here for file

## References

[B1] Manolio TA Collins FS Cox NJ Goldstein DB Hindorff LA Hunter DJ McCarthy MI Ramos EM Cardon LR Chakravarti A Finding the missing heritability of complex diseases Nature 2009 8 747 753 10.1038/nature08494 19812666 PMC2831613

[B2] Amiel J Sproat-Emison E Garcia-Barcelo M Lantieri F Burzynski G Borrego S Pelet A Arnold S Miao X Griseri P Hirschsprung disease, associated syndromes and genetics: a review J Med Genet 2008 8 1 14 10.1136/jmg.2007.055129 17965226

[B3] Borrego S Ruiz-Ferrer M Fernandez RM Antinolo G Hirschsprung’s disease as a model of complex genetic etiology Histol Histopathol 2013 8 1117 1136 23605783 10.14670/HH-28.1117

[B4] Borrego S Wright FA Fernandez RM Williams N Lopez-Alonso M Davuluri R Antinolo G Eng C A founding locus within the RET proto-oncogene may account for a large proportion of apparently sporadic Hirschsprung disease and a subset of cases of sporadic medullary thyroid carcinoma Am J Hum Genet 2003 8 88 100 10.1086/345466 12474140 PMC420016

[B5] Borrego S Ruiz A Saez ME Gimm O Gao X Lopez-Alonso M Hernandez A Wright FA Antinolo G Eng C RET genotypes comprising specific haplotypes of polymorphic variants predispose to isolated Hirschsprung disease J Med Genet 2000 8 572 578 10.1136/jmg.37.8.572 10922382 PMC1734658

[B6] Emison ES Garcia-Barcelo M Grice EA Lantieri F Amiel J Burzynski G Fernandez RM Hao L Kashuk C West K Differential contributions of rare and common, coding and noncoding Ret mutations to multifactorial Hirschsprung disease liability Am J Hum Genet 2010 8 60 74 10.1016/j.ajhg.2010.06.007 20598273 PMC2896767

[B7] Emison ES McCallion AS Kashuk CS Bush RT Grice E Lin S Portnoy ME Cutler DJ Green ED Chakravarti A A common sex-dependent mutation in a RET enhancer underlies Hirschsprung disease risk Nature 2005 8 857 863 10.1038/nature03467 15829955

[B8] Fernandez RM Boru G Pecina A Jones K Lopez-Alonso M Antinolo G Borrego S Eng C Ancestral RET haplotype associated with Hirschsprung’s disease shows linkage disequilibrium breakpoint at -1249 J Med Genet 2005 8 322 327 10.1136/jmg.2004.023960 15805159 PMC1736040

[B9] Bolk S Pelet A Hofstra RM Angrist M Salomon R Croaker D Buys CH Lyonnet S Chakravarti A A human model for multigenic inheritance: phenotypic expression in Hirschsprung disease requires both the RET gene and a new 9q31 locus Proc Natl Acad Sci U S A 2000 8 268 273 10.1073/pnas.97.1.268 10618407 PMC26652

[B10] Carrasquillo MM McCallion AS Puffenberger EG Kashuk CS Nouri N Chakravarti A Genome-wide association study and mouse model identify interaction between RET and EDNRB pathways in Hirschsprung disease Nat Genet 2002 8 237 244 10.1038/ng998 12355085

[B11] Gabriel SB Salomon R Pelet A Angrist M Amiel J Fornage M Attie-Bitach T Olson JM Hofstra R Buys C Segregation at three loci explains familial and population risk in Hirschsprung disease Nat Genet 2002 8 89 93 11953745 10.1038/ng868

[B12] Lin S Chakravarti A Cutler DJ Exhaustive allelic transmission disequilibrium tests as a new approach to genome-wide association studies Nat Genet 2004 8 1181 1188 10.1038/ng1457 15502828

[B13] Tang CS Sribudiani Y Miao XP de Vries AR Burzynski G So MT Leon YY Yip BH Osinga J Hui KJ Fine mapping of the 9q31 Hirschsprung’s disease locus Hum Genet 2010 8 675 683 10.1007/s00439-010-0813-8 20361209 PMC2871095

[B14] Garcia-Barcelo MM Fong PY Tang CS Miao XP So MT Yuan ZW Li L Guo WH Liu L Wang B Mapping of a Hirschsprung’s disease locus in 3p21 Eur J Hum Genet 2008 8 833 840 10.1038/ejhg.2008.18 18285831

[B15] Brooks AS Leegwater PA Burzynski GM Willems PJ de Graaf B van Langen I Heutink P Oostra BA Hofstra RM Bertoli-Avella AM A novel susceptibility locus for Hirschsprung’s disease maps to 4q31.3-q32.3 J Med Genet 2006 8 e35 16816022 10.1136/jmg.2005.038125 PMC2564564

[B16] Garcia-Barcelo MM Tang CS Ngan ES Lui VC Chen Y So MT Leon TY Miao XP Shum CK Liu FQ Genome-wide association study identifies NRG1 as a susceptibility locus for Hirschsprung’s disease Proc Natl Acad Sci U S A 2009 8 2694 2699 10.1073/pnas.0809630105 19196962 PMC2650328

[B17] Fernandez RM Bleda M Nunez-Torres R Medina I Luzon-Toro B Garcia-Alonso L Torroglosa A Marba M Enguix-Riego MV Montaner D Four new loci associations discovered by pathway-based and network analyses of the genome-wide variability profile of Hirschsprung’s disease Orphanet J Rare Dis 2012 8 103 10.1186/1750-1172-7-103 23270508 PMC3575329

[B18] Wang K Li M Bucan M Pathway-based approaches for analysis of genomewide association studies Am J Hum Genet 2007 8 1278 1283 10.1086/522374 17966091 PMC2276352

[B19] Wang K Li M Hakonarson H Analysing biological pathways in genome-wide association studies Nat Rev Genet 2010 8 843 854 10.1038/nrg2884 21085203

[B20] Fridley BL Biernacka JM Gene set analysis of SNP data: benefits, challenges, and future directions Eur J Hum Genet 2011 8 837 843 10.1038/ejhg.2011.57 21487444 PMC3172936

[B21] Aulchenko YS Ripatti S Lindqvist I Boomsma D Heid IM Pramstaller PP Penninx BW Janssens AC Wilson JF Spector T Loci influencing lipid levels and coronary heart disease risk in 16 European population cohorts Nat Genet 2009 8 47 55 10.1038/ng.269 19060911 PMC2687074

[B22] Askland K Read C Moore J Pathways-based analyses of whole-genome association study data in bipolar disorder reveal genes mediating ion channel activity and synaptic neurotransmission Hum Genet 2009 8 63 79 10.1007/s00439-008-0600-y 19052778

[B23] Torkamani A Topol EJ Schork NJ Pathway analysis of seven common diseases assessed by genome-wide association Genomics 2008 8 265 272 10.1016/j.ygeno.2008.07.011 18722519 PMC2602835

[B24] Purcell S Neale B Todd-Brown K Thomas L Ferreira MA Bender D Maller J Sklar P de Bakker PI Daly MJ Sham PC PLINK: a tool set for whole-genome association and population-based linkage analyses Am J Hum Genet 2007 8 559 575 10.1086/519795 17701901 PMC1950838

[B25] Medina I Montaner D Bonifaci N Pujana MA Carbonell J Tarraga J Al-Shahrour F Dopazo J Gene set-based analysis of polymorphisms: finding pathways or biological processes associated to traits in genome-wide association studies Nucleic Acids Res 2009 8 W340 W344 10.1093/nar/gkp481 19502494 PMC2703970

[B26] Medina I Carbonell J Pulido L Madeira SC Goetz S Conesa A Tarraga J Pascual-Montano A Nogales-Cadenas R Santoyo J Babelomics: an integrative platform for the analysis of transcriptomics, proteomics and genomic data with advanced functional profiling Nucleic Acids Res 2010 8 W210 W213 10.1093/nar/gkq388 20478823 PMC2896184

[B27] Benjamini Y Hochberg Y Controlling the false discovery rate: a practical and powerful approach to multiple testing J R Stat Soc Ser B 1995 8 289 300

[B28] Garcia-Alonso L Alonso R Vidal E Amadoz A de Maria A Minguez P Medina I Dopazo J Discovering the hidden sub-network component in a ranked list of genes or proteins derived from genomic experiments Nucleic Acids Res 2012 8 e158 10.1093/nar/gks699 22844098 PMC3488210

[B29] Minguez P Dopazo J Assessing the biological significance of gene expression signatures and co-expression modules by studying their network properties PLoS One 2011 8 e17474 10.1371/journal.pone.0017474 21408226 PMC3049771

[B30] Minguez P Gotz S Montaner D Al-Shahrour F Dopazo J SNOW, a web-based tool for the statistical analysis of protein-protein interaction networks Nucleic Acids Res 2009 8 W109 W114 10.1093/nar/gkp402 19454602 PMC2703972

[B31] Ward LD Kellis M HaploReg: a resource for exploring chromatin states, conservation, and regulatory motif alterations within sets of genetically linked variants Nucleic Acids Res 2012 8 D930 D934 10.1093/nar/gkr917 22064851 PMC3245002

[B32] Bernstein BE Birney E Dunham I Green ED Gunter C Snyder M An integrated encyclopedia of DNA elements in the human genome Nature 2012 8 57 74 10.1038/nature11247 22955616 PMC3439153

[B33] Liu JZ McRae AF Nyholt DR Medland SE Wray NR Brown KM Hayward NK Montgomery GW Visscher PM Martin NG Macgregor S A versatile gene-based test for genome-wide association studies Am J Hum Genet 2010 8 139 145 10.1016/j.ajhg.2010.06.009 20598278 PMC2896770

[B34] Mirina A Atzmon G Ye K Bergman A Gene size matters PLoS One 2012 8 e49093 10.1371/journal.pone.0049093 23152854 PMC3494661

[B35] Davydov EV Goode DL Sirota M Cooper GM Sidow A Batzoglou S Identifying a high fraction of the human genome to be under selective constraint using GERP++ PLoS Comput Biol 2010 8 e1001025 10.1371/journal.pcbi.1001025 21152010 PMC2996323

[B36] Garber M Guttman M Clamp M Zody MC Friedman N Xie X Identifying novel constrained elements by exploiting biased substitution patterns Bioinformatics 2009 8 i54 i62 10.1093/bioinformatics/btp190 19478016 PMC2687944

[B37] Meulemans D Bronner-Fraser M Gene-regulatory interactions in neural crest evolution and development Dev Cell 2004 8 291 299 10.1016/j.devcel.2004.08.007 15363405

[B38] Lim J Hao T Shaw C Patel AJ Szabo G Rual JF Fisk CJ Li N Smolyar A Hill DE A protein-protein interaction network for human inherited ataxias and disorders of Purkinje cell degeneration Cell 2006 8 801 814 10.1016/j.cell.2006.03.032 16713569

[B39] Goehler H Lalowski M Stelzl U Waelter S Stroedicke M Worm U Droege A Lindenberg KS Knoblich M Haenig C A protein interaction network links GIT1, an enhancer of huntingtin aggregation, to Huntington’s disease Mol Cell 2004 8 853 865 10.1016/j.molcel.2004.09.016 15383276

[B40] Camargo LM Collura V Rain JC Mizuguchi K Hermjakob H Kerrien S Bonnert TP Whiting PJ Brandon NJ Disrupted in Schizophrenia 1 Interactome: evidence for the close connectivity of risk genes and a potential synaptic basis for schizophrenia Mol Psychiatry 2007 8 74 86 10.1038/sj.mp.4001880 17043677

[B41] Soler-Lopez M Zanzoni A Lluis R Stelzl U Aloy P Interactome mapping suggests new mechanistic details underlying Alzheimer’s disease Genome Res 2011 8 364 376 10.1101/gr.114280.110 21163940 PMC3044851

[B42] Laranjeira C Pachnis V Enteric nervous system development: recent progress and future challenges Auton Neurosci 2009 8 61 69 10.1016/j.autneu.2009.09.001 19783483

[B43] Heanue TA Pachnis V Expression profiling the developing mammalian enteric nervous system identifies marker and candidate Hirschsprung disease genes Proc Natl Acad Sci U S A 2006 8 6919 6924 10.1073/pnas.0602152103 16632597 PMC1458994

[B44] Hirschhorn JN Lohmueller K Byrne E Hirschhorn K A comprehensive review of genetic association studies Genet Med 2002 8 45 61 10.1097/00125817-200203000-00002 11882781

[B45] Todd JA Statistical false positive or true disease pathway? Nat Genet 2006 8 731 733 10.1038/ng0706-731 16804532

[B46] Cirulli ET Goldstein DB Uncovering the roles of rare variants in common disease through whole-genome sequencing Nat Rev Genet 2010 8 415 425 10.1038/nrg2779 20479773

