# Supplementary material for: Pathways systematically associated to Hirschsprung’s disease
Source: Orphanet J Rare Dis. 2013 Dec 2;8:187. doi: 10.1186/1750-1172-8-187 (PMC3879038; doi:10.1186/1750-1172-8-187)
Supplement: Additional file 1: Table S1 — Summary of the effects of the different steps of the quality control. [file 1750-1172-8-187-S1.doc]

**Additional Table 1**. Results of quality control steps.

|  | | | ***Nsp*** | | ***Sty*** | |
| --- | --- | --- | --- | --- | --- | --- |
|  | | | **SNPs** | **Samples** | **SNPs** | **Samples** |
| **SAMPLES** | **START** |  | 262265 | 291 individuals | 238305 | 277 individuals |
|  | **Incomplete families** | 1 or 2 individuals | - | 21 samples (11 families) | - | 37 samples (19 families) |
|  | **Missing calls in samples** | Samples > 5% missing | - | 6 samples (5 families) ⇒ 15 samples removed | - | 5 samples (3 families) ⇒ 9 samples removed |
|  | **Mendelian errors in samples** | Families > 5% | - | 0 samples (0 families) | - | 0 samples (0 families) |
| **SNPs** | **Missing calls in SNPs** | SNPs > 20% missing | 454 | - | 427 | - |
|  | **Hardy-Weinberg** | SNPs p.val < 10-5 in Unaffected | 1883 | - | 1647 | - |
|  | **MAF** | MAF < 0.005 (0.5%) | 22676 | - | 16582 | - |
|  | **Mendelian errors in SNPs** | SNPs > 20% | 0 | - | 0 | - |
|  | | **Removed** | 24977 unique SNPs + 702 from PLINK | 36 samples | 18617 unique SNPs + 604 from PLINK | 46 samples |
|  | | **FINAL** | **236586 SNPs** | **255 samples (85 trios)** | **219084 SNPs** | **231 samples (77 trios)** |
