# Supplementary figures and images for: Pathways systematically associated to Hirschsprung’s disease
Source: Orphanet J Rare Dis. 2013 Dec 2;8:187. doi: 10.1186/1750-1172-8-187 (PMC3879038; doi:10.1186/1750-1172-8-187)

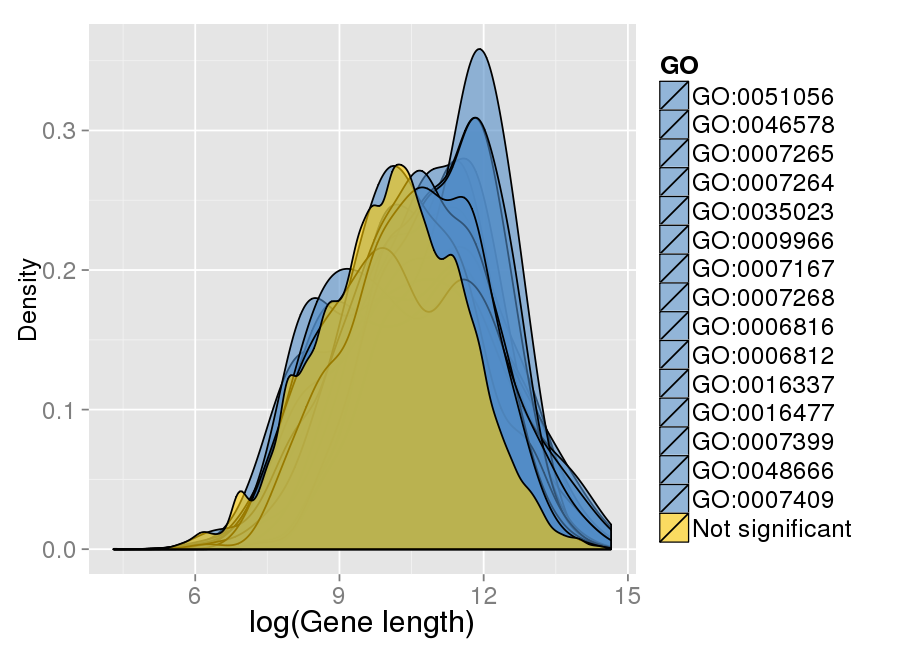

Supplement: Additional file 2: Figure S1 — Gene length distribution of gene lengths within GO terms. Significant GO terms from Table 2 are plotted in blue. The background distribution of gene lengths in the rest of non-significant GO terms is represented in yellow. [file 1750-1172-8-187-S2.png]

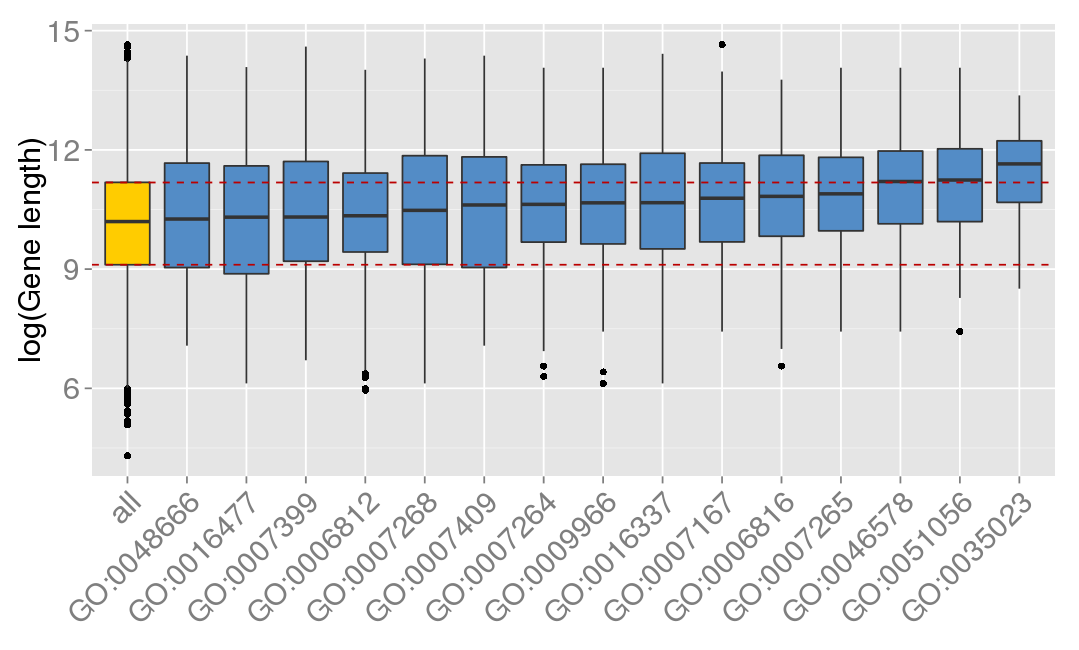

Supplement: Additional file 3: Figure S2 — Boxplots of gene length distribution of gene lengths within GO terms. The first boxplot on the left, in yellow, represents the distribution of genes in all the non-significant GO terms. The rest of boxplots in blue correspond to the significant GO terms from Table 2. [file 1750-1172-8-187-S3.png]

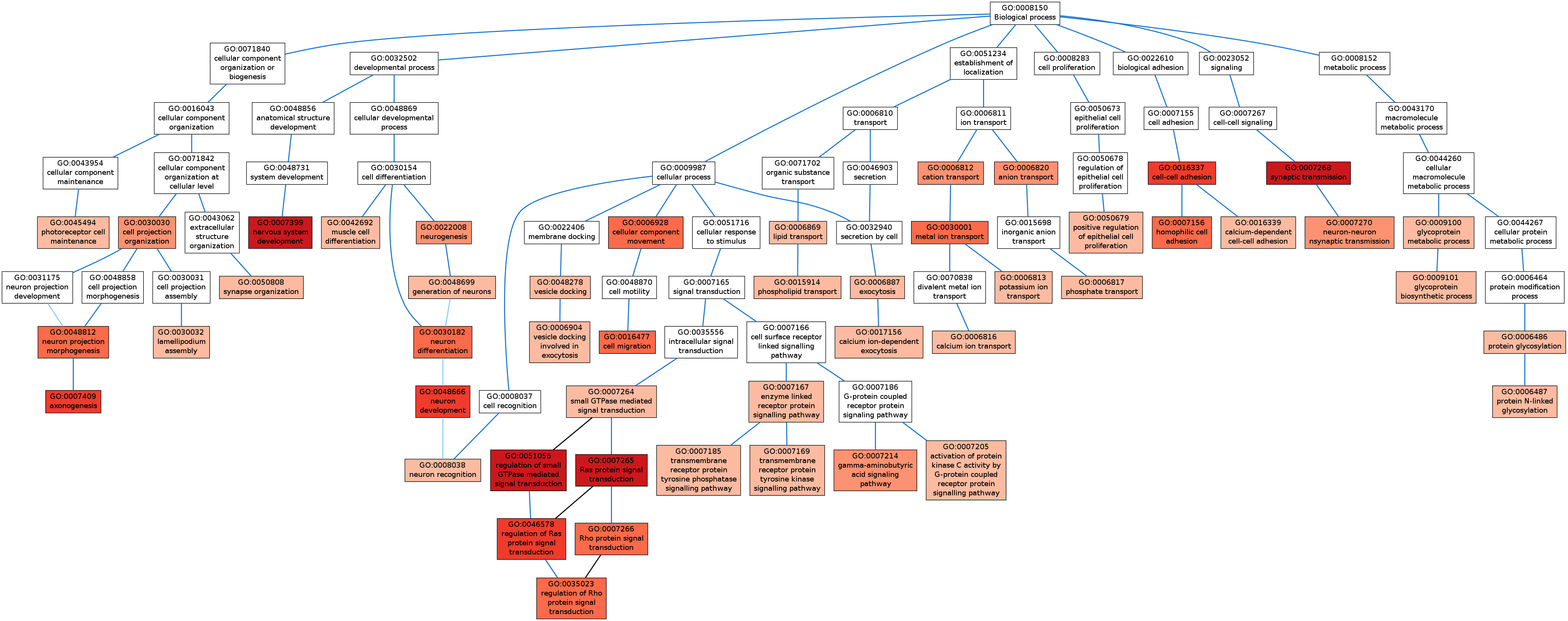

Supplement: Additional file 4: Figure S3 — Tree hierarchy depicting the relationships between GO terms significantly associated to HSCR (FDR adjusted p-value < 0.05) using the PBA [25] as implemented in Babelomics [26] in the four country populations of the Consortium: French, Italian, Dutch and USA for the Nsp chip. The results previously obtained for the Spanish population [17] have also been added. Significant terms have been color-coded according the number of populations in which the GO terms was found to be significant. The darkest values corresponds to GO terms significant in five populations and the palest in only one population (see Additional file 6: Table S2). [file 1750-1172-8-187-S4.png]

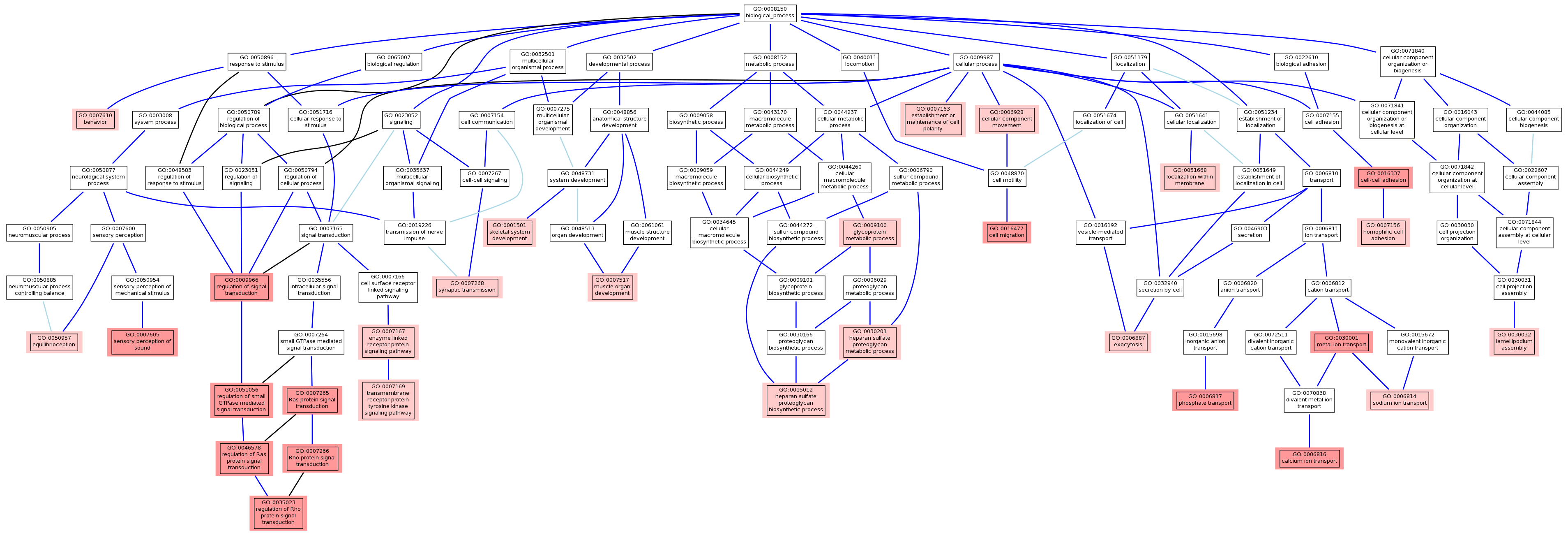

Supplement: Additional file 5: Figure S4 — Tree hierarchy depicting the relationships between GO terms significantly associated to HSCR (FDR adjusted p-value < 0.05) using the PBA [25] as implemented in Babelomics [26] in the four country populations of the Consortium: French, Italian, Dutch and USA for the Sty chip. The results previously obtained for the Spanish population [17] have also been added. Significant terms have been color-coded according the number of populations in which the GO terms was found to be significant. The darkest values corresponds to GO terms significant in two populations and the palest in only one population (see Additional file 7: Table S3). [file 1750-1172-8-187-S5.png]

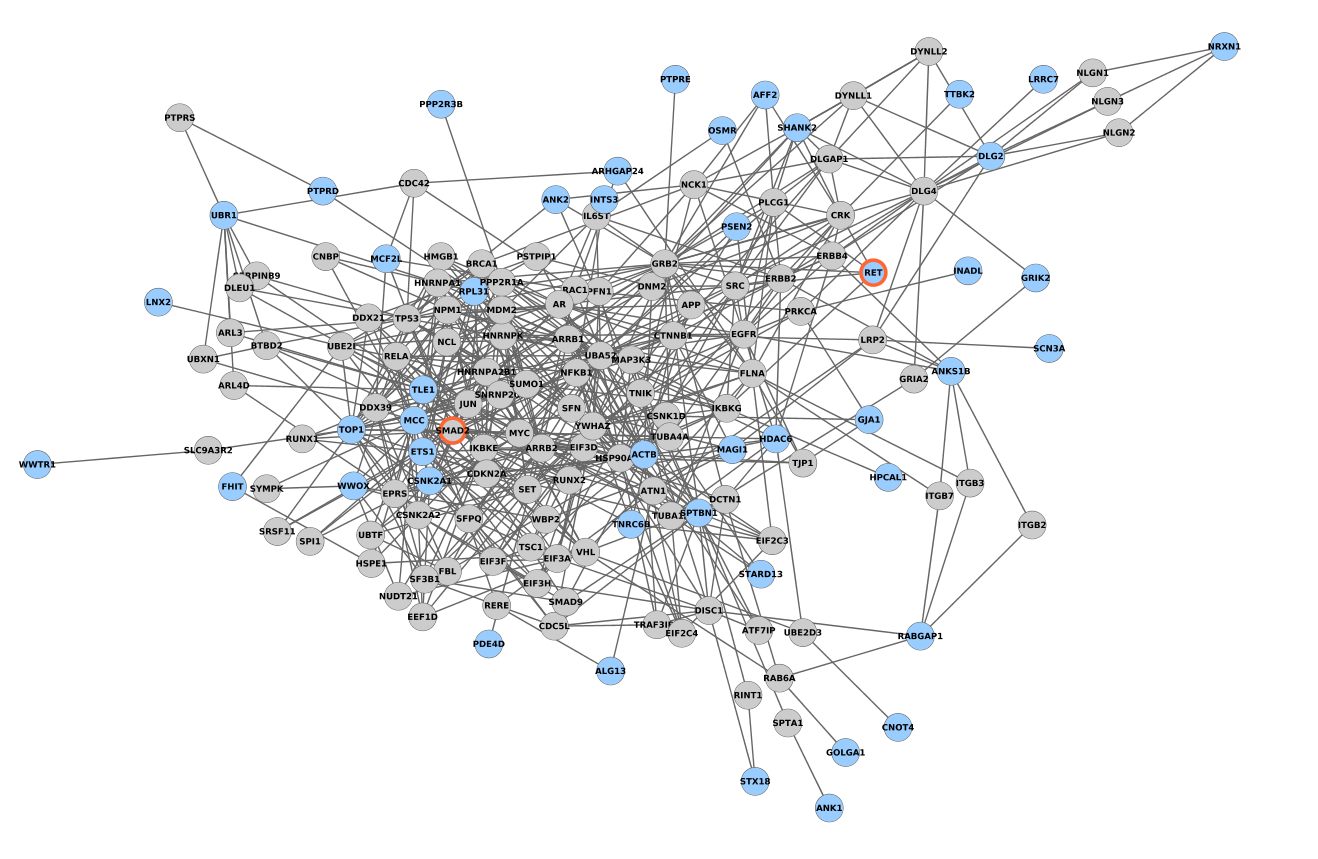

Supplement: Additional file 8: Figure S5 — Significant sub-network of 65 genes, allowing for one intermediate gene, associated to HSCR (p-value = 0.04) in the Sty chip. The network analysis [28] is carried out by using the functional analysis options (Set enrichment analysis/NetworkMiner) of Babelomics [26] on the list of genes ranked by the p-values obtained upon the application of a conventional TDT association test as implemented in PLINK [24]. [file 1750-1172-8-187-S8.png]
