# Supplementary material for: Pathways systematically associated to Hirschsprung’s disease
Source: Orphanet J Rare Dis. 2013 Dec 2;8:187. doi: 10.1186/1750-1172-8-187 (PMC3879038; doi:10.1186/1750-1172-8-187)
Supplement: Additional file 6: Table S2 — GO modules significantly associated to HSCR (FDR adjusted p-value < 0.05) using the PBA [25] as implemented in Babelomics [26] found in the analysis individualized by population in the Nsp chip. [file 1750-1172-8-187-S6.doc]

**Additional table 2**. GO modules significantly associated to HSCR (FDR adjusted p-value < 0.05) found in the analysis of the NSP chip individualized by population.

| **GO Term** | **Number of populations** | **Definition of GO term** |
| --- | --- | --- |
| GO:0007268 | 5 | synaptic transmission |
| GO:0051056 | 5 | regulation of small GTPase mediated signal transduction |
| GO:0007265 | 5 | Ras protein signal transduction |
| GO:0007399 | 5 | nervous system development |
| GO:0046578 | 4 | regulation of Ras protein signal transduction |
| GO:0048666 | 4 | neuron development |
| GO:0016337 | 4 | cell-cell adhesion |
| GO:0007409 | 4 | axonogenesis |
| GO:0007266 | 3 | Rho protein signal transduction |
| GO:0035023 | 3 | regulation of Rho protein signal transduction |
| GO:0048812 | 3 | neuron projection morphogenesis |
| GO:0030182 | 3 | neuron differentiation |
| GO:0030001 | 3 | metal ion transport |
| GO:0007156 | 3 | homophilic cell adhesion |
| GO:0006928 | 3 | cellular component movement |
| GO:0016477 | 3 | cell migration |
| GO:0022008 | 2 | neurogenesis |
| GO:0007270 | 2 | nerve-nerve synaptic transmission |
| GO:0007214 | 2 | gamma-aminobutyric acid signaling pathway |
| GO:0030030 | 2 | cell projection organization |
| GO:0006812 | 2 | cation transport |
| GO:0006820 | 2 | anion transport |
| GO:0006904 | 1 | vesicle docking during exocytosis |
| GO:0048278 | 1 | vesicle docking |
| GO:0007185 | 1 | transmembrane receptor protein tyrosine phosphatase signaling pathway |
| GO:0007169 | 1 | transmembrane receptor protein tyrosine kinase signaling pathway |
| GO:0050808 | 1 | synapse organization |
| GO:0007264 | 1 | small GTPase mediated signal transduction |
| GO:0006487 | 1 | protein amino acid N-linked glycosylation |
| GO:0006486 | 1 | protein amino acid glycosylation |
| GO:0006813 | 1 | potassium ion transport |
| GO:0050679 | 1 | positive regulation of epithelial cell proliferation |
| GO:0045494 | 1 | photoreceptor cell maintenance |
| GO:0015914 | 1 | phospholipid transport |
| GO:0006817 | 1 | phosphate transport |
| GO:0008038 | 1 | neuron recognition |
| GO:0042692 | 1 | muscle cell differentiation |
| GO:0006869 | 1 | lipid transport |
| GO:0030032 | 1 | lamellipodium assembly |
| GO:0009100 | 1 | glycoprotein metabolic process |
| GO:0009101 | 1 | glycoprotein biosynthetic process |
| GO:0048699 | 1 | generation of neurons |
| GO:0006887 | 1 | exocytosis |
| GO:0007167 | 1 | enzyme linked receptor protein signaling pathway |
| GO:0015674 | 1 | di-, tri-valent inorganic cation transport |
| GO:0006816 | 1 | calcium ion transport |
| GO:0017156 | 1 | calcium ion-dependent exocytosis |
| GO:0016339 | 1 | calcium-dependent cell-cell adhesion |
| GO:0007205 | 1 | activation of protein kinase C activity by G-protein coupled receptor protein signaling pathway |
