# Supplementary material for: Pathways systematically associated to Hirschsprung’s disease
Source: Orphanet J Rare Dis. 2013 Dec 2;8:187. doi: 10.1186/1750-1172-8-187 (PMC3879038; doi:10.1186/1750-1172-8-187)
Supplement: Additional file 7: Table S3 — GO modules significantly associated to HSCR (FDR adjusted p-value < 0.05) using the PBA [25] as implemented in Babelomics [26] found in the analysis individualized by population in the Sty chip. [file 1750-1172-8-187-S7.doc]

**Additional table 3**. GO modules significantly associated to HSCR (FDR adjusted p-value < 0.05) found in the analysis of the STY chip individualized by population.

| **GO Term** | **Number of populations** | **Definition of GO term** |
| --- | --- | --- |
| GO:0007266 | 2 | Rho protein signal transduction |
| GO:0051056 | 2 | regulation of small GTPase mediated signal transduction |
| GO:0009966 | 2 | regulation of signal transduction |
| GO:0035023 | 2 | regulation of Rho protein signal transduction |
| GO:0007265 | 2 | Ras protein signal transduction |
| GO:0006817 | 2 | phosphate transport |
| GO:0030001 | 2 | metal ion transport |
| GO:0015674 | 2 | di-, tri-valent inorganic cation transport |
| GO:0016477 | 2 | cell migration |
| GO:0016337 | 2 | cell-cell adhesion |
| GO:0006816 | 2 | calcium ion transport |
| GO:0007169 | 1 | transmembrane receptor protein tyrosine kinase signaling pathway |
| GO:0007268 | 1 | synaptic transmission |
| GO:0006814 | 1 | sodium ion transport |
| GO:0001501 | 1 | skeletal system development |
| GO:0007517 | 1 | muscle organ development |
| GO:0051668 | 1 | localization within membrane |
| GO:0030032 | 1 | lamellipodium assembly |
| GO:0007156 | 1 | homophilic cell adhesion |
| GO:0030201 | 1 | heparan sulfate proteoglycan metabolic process |
| GO:0015012 | 1 | heparan sulfate proteoglycan biosynthetic process |
| GO:0009100 | 1 | glycoprotein metabolic process |
| GO:0006887 | 1 | exocytosis |
| GO:0007163 | 1 | establishment or maintenance of cell polarity |
| GO:0050957 | 1 | equilibrioception |
| GO:0007167 | 1 | enzyme linked receptor protein signaling pathway |
| GO:0006928 | 1 | cellular component movement |
| GO:0007610 | 1 | behavior |
| GO:0001501 | 1 | skeletal system development |
